# Severe deterioration in sperm parameters and testes of rats administered naproxen and diclofenac at pre-puberty: An experimental study

**DOI:** 10.18502/ijrm.v20i10.12267

**Published:** 2022-11-02

**Authors:** Tamadir H. W. Aledani, Manal N. Al-Hayder, Rawaa S. Al-Mayyahi

**Affiliations:** ^1^Department of Clinical Laboratory Sciences, College of Pharmacy, University of Basrah, Basrah, Iraq.; ^2^Department of Pharmacology and Toxicology, College of Pharmacy, University of Basrah, Basrah, Iraq.

**Keywords:** Male infertility, Nonsteroidal drugs, Pathology, Spermatozoa, Testis.

## Abstract

**Background:**

Although naproxen and diclofenac are the most commonly used nonsteroidal drugs, their toxicity affecting male reproductivity has not been sufficiently studied, particularly when used in pre-puberty.

**Objective:**

This study aims to investigate the toxic effects of naproxen and diclofenac on sperm parameters and testes in pre-pubertal rats.

**Materials and Methods:**

In this experimental study, 15 pre-pubertal male albino rats (aged 5 wk, weighted 70-80 gr) were used. The animals were divided in to 3 groups (n = 5/each) of control (0.1 ml dimethyl sulfoxide), naproxen (50 mg/kg), and diclofenac sodium (5 mg/kg), and were orally administered with these drugs every day for 3 wk. Epididymal spermatozoa were taken to assess sperm count, viability, and morphology. After preparing tissue sections, testicular histopathological and histomorphometric analyses were performed.

**Results:**

The body weights of rats in both naproxen and diclofenac groups significantly decreased (p < 0.001 and p = 0.03, respectively) in comparison to the control group. Remarkably, the testis weight and total sperm number significantly decreased (p = 0.002, and p = 0.004 respectively) in the naproxen-administered rats only. The sperm viability percentage decreased significantly in both diclofenac and naproxen-administered groups (p ≤ 0.001 and p = 0.03 respectively). Moreover, there was a significant increase (p < 0.001) in the percentage of sperm morphological anomalies in both drug-administered groups. Also, the histological and morphometric findings exhibited severe histopathological appearances in the testes and seminiferous tubules parameters in both drug-administered groups.>

**Conclusion:**

Naproxen and diclofenac administrations of rats before their puberty induce considerable harm to sperm parameters and testicular histology and morphometry. These severe toxic effects can lead to potential infertility.

## 1. Introduction

Spermatogenesis is a continuous process to produce sperm during the reproductive lifetime. The spermatozoa generate and develop in the seminiferous tubules of testes from spermatogenic stem cells that are called spermatogonia. The generation of normal and mobile gametes is an imperious necessity for male fertility (1).

Many reasons can impact spermatogenesis and sperm maturation, and these can lead to male infertility. Using therapeutic drugs is among the major causes that can harm spermatogonia and spermatogenesis (2). Nonsteroidal anti-inflammatory drugs are broadly used to treat inflammatory diseases such as osteoarthritis, rheumatoid arthritis, etc. (3).

The wide-ranging risks of using these nonsteroidal drugs are serious cardiovascular, gastrointestinal, and renal damage (4). However, little is known about their effects on the reproductive system. It is noteworthy that nonsteroidal anti-inflammatory drugs act as inhibitors of the cyclooxygenases, which can stimulate prostaglandins production such as prostaglandin E ${}_{2}$ . These prostaglandins are important for many physiological functions and they regulate diverse processes (5). Many studies refer to the important role of prostaglandins in ovulation; in addition their inhibition may lead to female infertility through the chronic use of nonsteroidal drugs (6-8).

For males, the prostaglandins are produced by 2 somatic cells (Leydig and Sertoli cells) in the testes and they have critical roles in spermatogenesis, steroidogenesis, and regulation of the spermatogenic competence (9). Furthermore, cyclooxygenase-2 is highly expressed in the testis, epididymis, and spermatozoa of boars; it may play an effective role in the sperms quality and fertility (10). Thus, drugs that inhibit cyclooxygenases can impair male fertility. Although naproxen and diclofenac are the most commonly used of nonsteroidal anti-inflammatory drugs, there are no sufficient studies on their toxicity affecting male fertility particularly when they are used in the period before sexual maturation.

Consequently, the current study aims to investigate the toxic effects of naproxen and diclofenac sodium on sperm parameters and testes in male rats when they are administered during their pre-pubertal period.

## 2. Materials and Methods

### Study design and drugs administration

To achieve the goal of this experimental study, 15 pre-pubertal male albino Wistar-Kyoto rats aged 5 wk (70-80 gr) were used and housed in the animals' house of Basrah University, Basrah, Iraq where there were controlled suitable conditions for housing the animals (12 hr light/dark cycle, and 25 C).

Rats were divided into 3 groups (n = 5/each) and administered by oral gavage every day (in the morning, once per day): A) control rats were administered with 0.1 ml of the dimethyl sulfoxide (5%) (ROMIL LTD, United Kingdom), B) rats were administered with 50 mg/kg body weight of naproxen (PiONEER, IRAQ), and C) rats were administered with 5 mg/kg of diclofenac sodium (MICRO LABS LIMITED, INDIA) respectively. The dimethyl sulfoxide was used to dissolve naproxen and diclofenac sodium. The drugs administration continued for 3 wk (11). At the end of administration, the animals were firstly weighed and then anaesthetized to prevent pain and distress through refinement. After that they were dissected. Subsequently, the testes and epididymides were carefully removed and also weighed to be used in subsequent investigations.

###  Evaluation of the sperm parameters

For sperm analysis, the epididymis of each rat was kept in 10 ml of Hibernate HE-Ca medium (Brain Bit, UK). Then the epididymis was teared to leak the sperms into the medium. The World Health Organization protocol was followed to assess sperm count, viability, and morphology as described before (12) with modifications. Briefly, total sperm number per ml was estimated by counting the heads of sperm manually under a light microscope (Genex, USA) using a hemocytometer after preparing a dilution of the sperm-medium suspension (1 ml) with formaldehyde fixative (9 ml).

For sperm viability and morphology, trypan blue staining (Beijing Solaria science, China) was utilized and was prepared as a stock solution (0.4% trypan blue in phosphate-buffered saline, pH 7.3). Then, one volume of this stock was mixed with equal volume of sperm-medium suspension to be used immediately for microscopic examination (400 × ). A total number of 200 spermatozoa were counted for each rat. The trypan blue stained slides were air-dried and then used to detect sperm anomalies. The percentage of sperm viability and morphological abnormalities were calculated as: 


$$Spermviability\%=\frac{viablesperms\left(unstained\right)}{viablesperms+nonviablesperms\left(stainedblue\right)}\times 100$$

$$Spermmorphologicalanomalies\%=\frac{abnormalsperms}{abnormalsperms+normalsperms}\times 100$$

### Histopathological and histomorphometric analyses of the testes

After excising the testes from the dissected animals, they were fixed in 10% formalin; then were passed into a series of alcohols and cleared with xylene. Subsequently, they were embedded in paraffin wax. A rotary microtome (Bioevopeak, China) was used to obtain tissue sections thickened at 5 μm. These sections were stained using hematoxylin and eosin stain for histological investigations. For histomorphometric analysis, the average diameter of seminiferous tubular and their lumen were performed by randomly measuring 10 circular tubules in the transverse sections for each animal. In the same sections, the thickness of the seminiferous epithelium (from the basement membrane to the tubular lumen) was measured. All the histomorphometric parameters mentioned above were conducted using digital images of 200 × magnification with the software ImageJ (National Institutes of Health, USA).

### Ethical considerations

The current study was approved by Basrah University Ethical Committee for animal research, Basrah, Iraq (Code: 3108003). The ethical guidelines for caring and working with laboratory animals were precisely followed. The origin of the healthy animals (had no genetic modification) was from College of Veterinary Medicine, University of Basrah.

### Statistical analysis

GraphPad software (California, USA) was conducted to statistically analyse the results by using one-way analysis of variance (ANOVA) and Bonferroni's post-test. P-values < 0.05 were determined as significant values represented by mean ± SEM.

## 3. Results

### Effect of the drugs on body and testis weights

**Figure 1:**
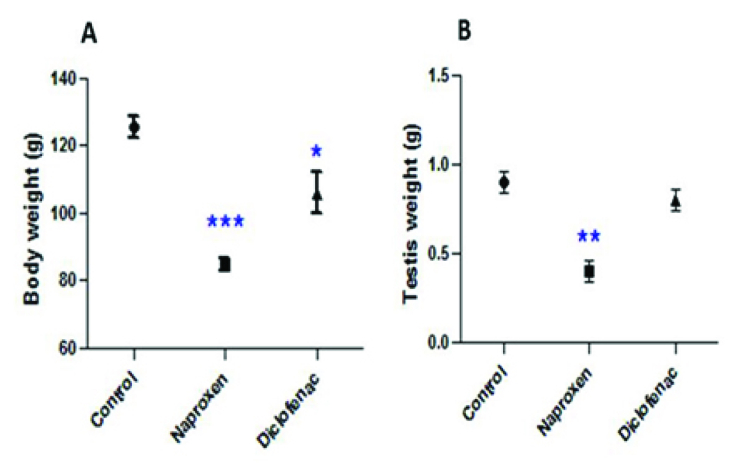
Decline in the body and testis weights induced by nonsteroidal drugs administration versus the control. (A) Significant decline in the bodyweight caused by both naproxen and diclofenac sodium. (B) Reduction in the testis weight. *p = 0.03, **p = 0.002, *** p < 0.001.

Both naproxen and diclofenac sodium administrations significantly reduced the body weights of the albino rats (p < 0.001 and p = 0.03 respectively) (Figure 1A). The testis weight significantly decreased in the naproxen-administered rats (p = 0.002) compared to the diclofenac sodium-administered rats, which exhibited a nonsignificant decrease in the testis weight (Figure 1B).

### Alteration of the sperm parameters

**Figure 2:**
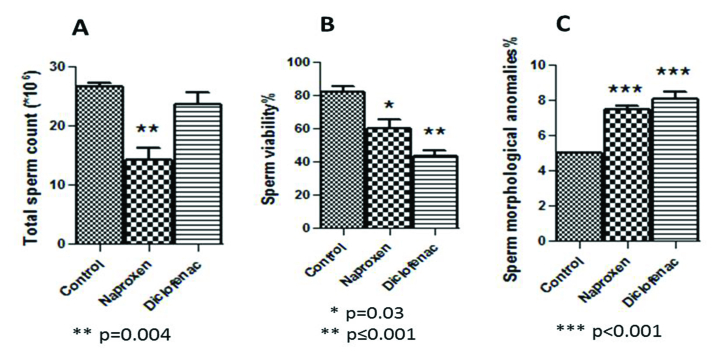
Abnormal alterations of sperm parameters resulted in the rats being administered nonsteroidal drugs vs. the control. (A) Changes in the total sperm count values. (B) Significantly changed values of the sperm viability percentage. (C) Significant values of sperm abnormalities.

**Figure 3:**
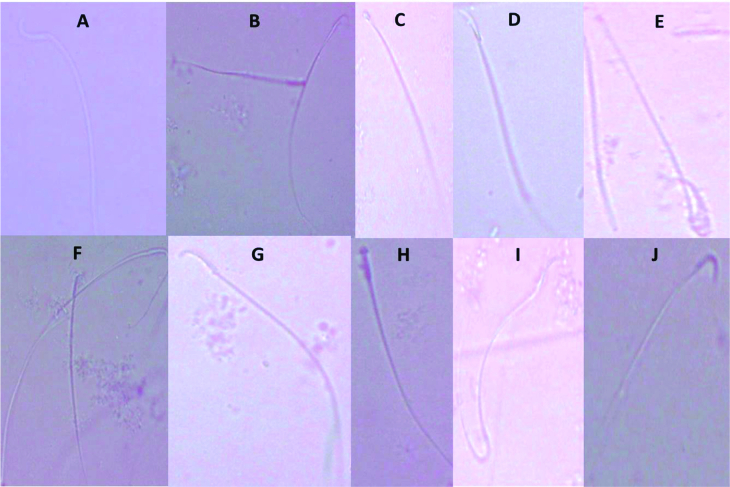
Sperm anomalies induced in rats administered naproxen and diclofenac sodium compared with the control rats. (A) Normal sperm in the control rats, (B) Sperm with double tails and amorphous head, (C) Sperm with small and amorphous head, (D) Sperm with banana-shaped head, (E) Sperm with folded tail and without a head, (F) Sperm with flattened or reduced head, (G) Sperm with short hook, (H) Sperm with pinhead, (I) Sperm having no hook with a bent tail, and (J) Sperm with a bent neck. Magnification: 400X.

**Figure 4:**
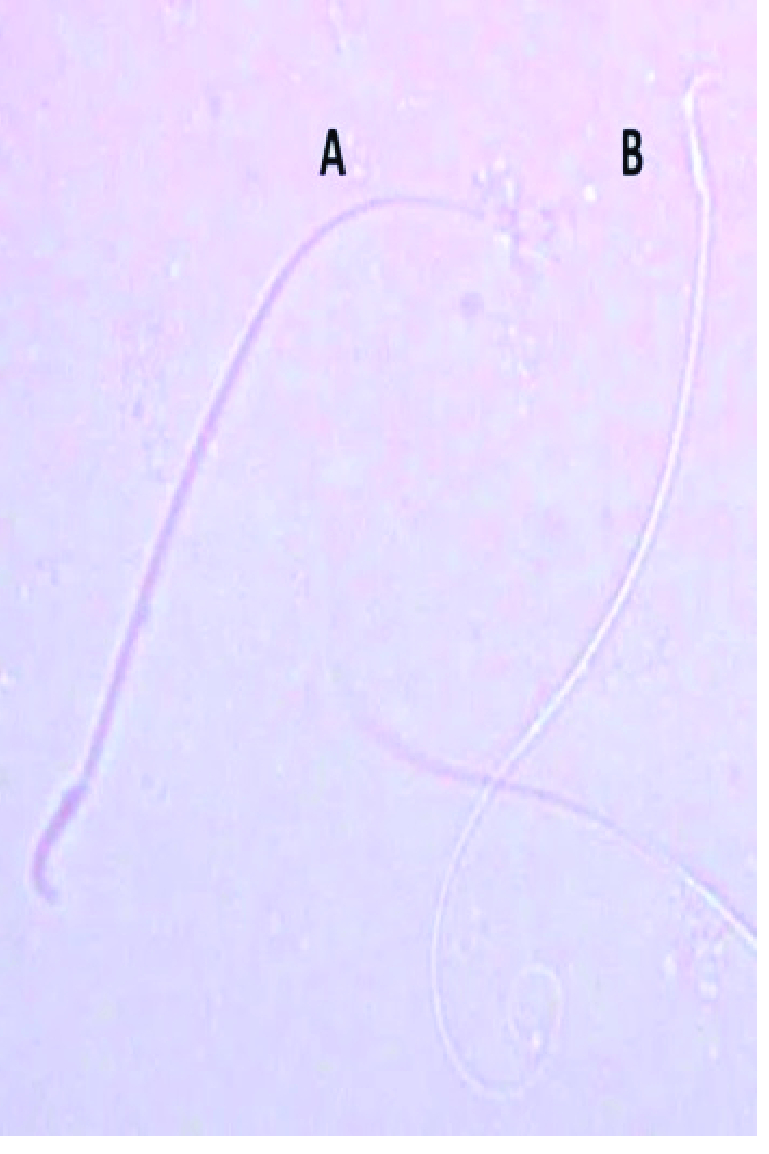
Epididymal sperms of the rats stained with trypan blue (400X). (A) Nonviable sperm stained blue. (B) Colorless viable sperm.

Figure 2 showed a significant decline in the total number of epididymal spermatozoa in the naproxen-administered rats (p = 0.004) in comparison to the control rats. But, sperm count decreased insignificantly in the diclofenac sodium-administered group (Figure 2A). On the other hand, the sperm viability percentage was significantly diminished in both diclofenac and naproxen-administered groups (p ≤ 0.001 and p = 0.03 respectively) (Figure 2B). Also, in both drug-administered groups, there was a significant increase (p < 0.001) in the percentage of sperm morphological anomalies (Figure 2C). These sperm anomalies were shown in figure 3, including sperm with double tails and amorphous head, small and amorphous head, banana-shaped head, folded tail and without a head, flattened or reduced head, short hook, pinhead, no hook with a bent tail, and bent neck. Notably, the nonviable sperms stained blue were differentiated from the viable sperms that were unstained, as shown distinctly in figure 4.

### Testicular histopathology and histomorphometry

**Figure 5:**
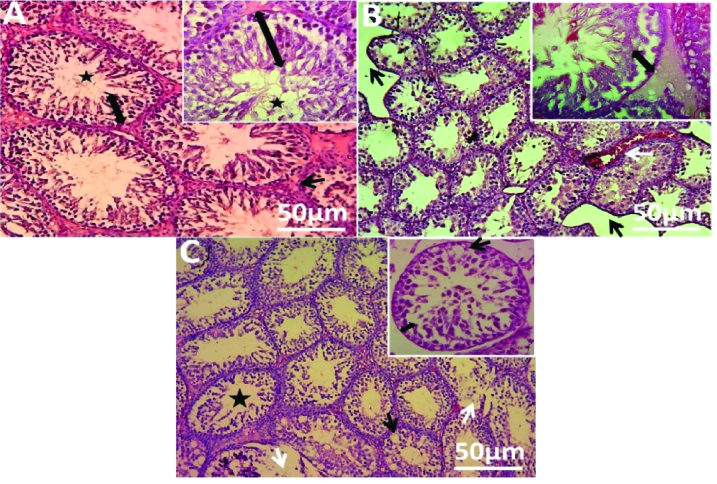
Histopathological alterations in the rats' testes following nonsteroidal drugs administration versus the control. (A) Normal seminiferous tubule with sperm (asterisk) lined by germinal epithelium (double arrow) and surrounded by interstitial connective tissue (black arrow) in the control group. (B) Separation of spermatogenic cells (double arrow) and loss of interstitial connective tissue (black arrow) with foci of the congestion (white arrow) in the naproxen-administered group. (C) Degeneration in several seminiferous tubules (white arrow), the wide lumen of seminiferous tubules with few sperms (asterisk), and clear vacuolization (black arrow) in diclofenac sodium-administered group. Stain: Haematoxylin & eosin. Magnifications of main images: 200X; inset: 400X.

Histological analysis of the present study showed that the control group revealed normal testicular parenchymal architecture with compactly arranged seminiferous tubules at all spermatogenesis stages and interstitial cells (Figure 5A). Naproxen-administered group exhibited clear vacuolization, separation of spermatogenic cells, and loss of interstitial connective tissue, which was replaced with a few foci of congestions (Figure 5B). Also, the diclofenac sodium-administered group showed signs of degeneration in several seminiferous tubules, reduction in the number of spermatogonial cells, and clear vacuolization (Figure 5C).

**Figure 6:**
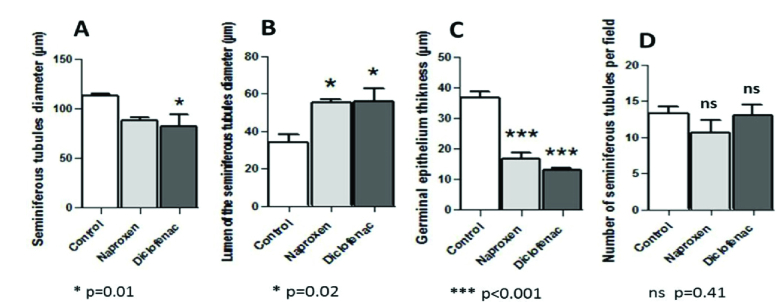
Morphometric alterations of seminiferous tubules in the rat testes resulted from nonsteroidal drugs administration vs. the control. (A) Decrease in the diameter of seminiferous tubules. (B) Significant increase in the diameter of lumen tubules. (C) Significant reduction in the thickness of the germinal epithelium. (D) Nonsignificant decrease in the number of seminiferous tubules per field.

The morphometric findings of seminiferous tubules compared to the control revealed that diameters of the tubules decreased in both drug-administered groups. However, this reduction was significant in the diclofenac sodium-administered group only (p = 0.01) (Figure 6A). Diameters of the tubule lumens, on the other hand, appeared to have a significant increase (p = 0.02) in both drug-administered groups (Figure 6B). Further, in both drug groups, a significant reduction (p < 0.001) was observed in the thickness of the germinal epithelium of the tubules (Figure 6C). Otherwise, the number of the seminiferous tubules per field revealed no significant differences (p = 0.41) between the control and nonsteroidal drug-administered groups (Figure 6D).

## 4. Discussion

Our results demonstrated the harmful effects of both naproxen and diclofenac sodium as nonsteroidal drugs on sperm parameters and testicular histology that may lead to infertility. The drugs administration at the pre-pubertal period of the rats could impact sperm maturation and thereby causing male infertility. Male fertility can be affected when drugs induce harm or damage in the testes and impair spermatogenesis and sperm maturation (2). The harmful effects of naproxen in this study were represented by a significant decrease in the sperm count and seminiferous tubules injuries; such results were similar to those obtained in a previous study (13). Remarkably, there were no more available studies concerning the reproductive toxicity of naproxen in the rats such as the percentage of sperm viability reduction and the percentage of increasing sperm morphological abnormalities. In this context, the previous studies reported a reduction in the total sperm count and sperm viability percentage caused by diclofenac sodium and that corresponds with our results. As for the histopathological effects resulting from diclofenac sodium administration, the aforementioned studies recorded almost similar effects, including degeneration and shrinkage of the seminiferous tubules, vacuolization in the interstitial connective tissue, and depletion of germinal epithelium (14-16).

According to our findings, the degeneration and depletion of the germinal layer mediated by testicular damage might be the reason for the decrease of spermatogonial cells that led to a reduction in sperm number and subsequently affected fertility. The cause of increased sperm anomalies induced by both naproxen and diclofenac sodium might be due to the presence of nuclear DNA damage, or fragmentation in the spermatozoa that is considered the main pathway responsible for sperm morphological defects, as well as chromatin immaturity (17). There is a correlation between the DNA fragmentation and morphological defects of sperms in patients with teratozoospermia (18).

Also, the presence of apoptotic markers in spermatozoa have been strongly related to sperm morphological abnormalities (19). Increasing the apoptosis level during early spermatogenesis results in sperm apoptosis that leads to male infertility (20). Sperm DNA damage and apoptosis can be created by excessive oxidative stress. Oxidative stress has also been correlated with abnormal sperm morphology (19). Moreover, oxidative stress can also reduce sperm viability, and vitamin E administration as an antioxidant leads to reverse this effect significantly (12). It is worth mentioning that our findings of deteriorated sperm parameters (sperm count, viability, and morphology) and injured seminiferous tubules caused by naproxen and diclofenac sodium were in parallel with the toxic effects of other reproductive toxicants such as sodium arsenite and manganese dioxide micro- and nanoparticles (12, 21).

With regards to the morphometric outcomes, the present study showed a decrease in the seminiferous tubules' diameters and their germinal epithelium thickness in both naproxen and diclofenac sodium-administered groups. These findings were in accordance with the toxic effects of zinc oxide nanoparticles in mouse testis and di-butyl phthalate-induced oxidative stress in the testis of rats (22, 23). The reduction in seminiferous diameter may indicate increased loss of the germ cells through apoptosis (22). Also, the spermatogenesis process's rise results in an expansion of the seminiferous tubules' diameter and thickness (24). Thus, the histomorphological alterations in our study might be attributed to the reduction in the number of spermatogonial cells and spermatogenesis. Furthermore, the significantly increased diameters of tubule lumens were manifested in both drug-administered groups of the present study. The larger lumen and thinner epithelium of the seminiferous tubules were suggested to be an evidence of increased intra-tubular pressure-mediated spermatogenic damage (25).

It is noteworthy that the body weights of rats administered the nonsteroidal drugs significantly declined compared to the control rats. Researchers (14) mentioned that the diclofenac sodium treatment can reduce feed intake and body weight. As for naproxen, it is considered an inhibitor of glycogen synthase kinase-3β, and this pharmacological inhibitor caused a significant reduction in the weights of obese mice (26). Glycogen is the main origin for glucose and plays a crucial role in growing gonads during development (27). Also, inhibition of androgen biosynthesis may be indirectly responsible for significantly reducing the weights of the testis, epididymis, ventral prostate, and seminal vesicle caused by aspirin (nonsteroidal anti-inflammatory drug) treatment; as well as androgen hormones closely regulate the weight, size, and function of testes and other reproductive organs (28). These facts might be involved in the significant drop of testis weight; which was considerable in the naproxen-administered group. Otherwise, diclofenac sodium administration produced a nonsignificant decline in the testis weights. This insignificant decline did not agree with the results of a previous study (16).

## 5. Conclusion

It can be concluded from the current study that both naproxen and diclofenac sodium exert harms on the sperm parameters (sperm numbers, viability, and morphology), the testicular histology and morphometry in the albino rats when administered before their sexual maturity. These pathological effects are very deleterious to male fertility and can lead to potential infertility.

##  Acknowledgments

This study has not been supported financially from any organization or agency.

##  Conflict of Interest

The authors declare that there is no conflict of interest.
